# Editorial: New Insights into Microbial Ecology through Subtle Nucleotide Variation

**DOI:** 10.3389/fmicb.2016.01318

**Published:** 2016-08-24

**Authors:** A. Murat Eren, Mitchell L. Sogin, Loïs Maignien

**Affiliations:** ^1^Department of Medicine, The University of ChicagoChicago, IL, USA; ^2^Marine Biological Laboratory, Josephine Bay Paul CenterWoods Hole, MA, USA; ^3^Laboratory of Microbiology of Extreme Environnments, UMR 6197, Institut Européen de la Mer, Université de Bretagne OccidentalePlouzane, France

**Keywords:** oligotyping, minimum entropy decomposition, 16S rRNA gene, microbial ecology, high resolution

Characterizing the community structure of naturally occurring microbes through marker gene amplicons has gained widespread acceptance for profiling microbial populations. The 16S ribosomal RNA (rRNA) gene provides a suitable target for most studies since (1) it meets the criteria for robust markers of evolution, e.g., both conserved and rapidly evolving regions that do not undergo horizontal gene transfer, (2) microbial ecologists have identified widely adopted primers and protocols for generating amplicons for sequencing, (3) analyses of both cultivars and environmental DNA have generated well-curated databases for taxonomic profiling, and (4) bioinformaticians and computational biologists have published comprehensive software tools for interpreting the data and generating publication-ready figures. Since the initial descriptions of high-throughput sequencing of 16S rRNA gene amplicons to survey microbial diversity, we have witnessed an explosion of association-based inferences of interactions between microbes and their environment.

Despite these advances, the field of microbial ecology faces numerous technical challenges. Sampling and storage strategies, DNA extraction protocols, limitations of the so called “universal” PCR primers, random sequencing errors, and the identification of ecologically relevant units can bias interpretations of observations based on 16S rRNA gene data. Although microbiologists comprehend most of these challenges, the need for handling large number of sequences, and to partition these complex data into appropriate proxies for environmental genomes caught almost everyone off-guard.

*De novo* clustering of short reads into operational taxonomic units (OTUs) based on “pairwise sequence similarities” quickly became the primary way to partition sequencing data into ecological units as this approach significantly out-performed analyses that relied strictly upon taxonomy. On the other hand, as random sequencing errors can dramatically increase the number of mismatches between two aligned reads, the underlying principle of most *de novo* clustering algorithms that rely on the edit distance was prone to inflating the diversity estimations. The use of 97% sequence similarity threshold emerged as a *de facto* standard, and has successfully reduced the impact of erroneous OTUs on diversity estimations. However, the computational convenience this arbitrary threshold offers has been at the expense of accurate ecological inference, as 3% OTUs are often phylogenetically mixed, and inconsistent (Koeppel and Wu, 2013; Eren et al., 2014; Nguyen et al., 2016).

Oligotyping (Eren et al., 2013) proposes an alternative way to decompose marker gene amplicons. It first considers the entire sequencing data to identify variable nucleotide positions, and then utilizes only those positions that show significant variation to partition reads into oligotypes. The identification of variable nucleotide positions in the oligotyping workflow relies on Shannon entropy (Shannon, 1948), which is a measure of information uncertainty (Jost, 2006). The association between the measured entropy and the diversity of nucleotides at a given nucleotide position in a dataset of sequences allows the identification of nucleotide positions that likely carry phylogenetically important signal. The departure from pairwise sequence alignments, and the use of entropy-based decomposition strategy, makes it possible to resolve closely related but distinct taxa that differ by as little as one nucleotide at the sequenced region.

Our research topic contains original research and method papers that employs oligotyping of microbial community data to investigate ecological questions in divergent environments including the human oral cavity (Mark Welch et al.), mammalian guts (Menke et al.), deep-sea sediments (Buttigieg and Ramette), as well as freshwater (Newton and McLellan), sewage (Fisher et al.), marine (Delmont et al.), and soil (Turlapati et al.) ecosystems. In a study that cuts across multiple environments, Schmidt et al. uses oligotyping to investigate the Vibrio ecology in environmental, as well as host- and substrate-associated habitats. Ramette and Buttigieg implements an R package for entropy-based decomposition procedures, and their software library contains additional approaches, such as the “broken stick model” procedure to identify low-abundance oligotypes that could be generated by chance alone, and a “one-pass entropy profiling” approach to efficiently identify those OTUs whose decomposition into oligotypes would most likely explain concealed diversity (Ramette and Buttigieg). Finally, Utter et al. reconcile the individuality, stability, and variability of the oral microbial communities in the context of “spatial structure” of microbes in dental plaque by combining high-resolution depiction of microbial community data with high-resolution imaging of multi-taxa microbial consortia in the human oral cavity (Mark Welch et al., 2016).

Most articles in this collection demonstrate the importance of high-resolution analyses, and provide further evidence that reveals the need to open the “black box” of OTUs in microbial ecology. Doing so not only allows finer representation of the microbial diversity in a wide range of ecosystems, but also improves the ecological signal for downstream analyses that aim to infer correlations (McLellan and Eren, 2014; Reveillaud et al., 2014; Eren et al., 2015; Kleindienst et al., 2015).

While oligotyping demonstrates the efficacy of an entropy-based concept to partition closely related taxa, the algorithm minimum entropy decomposition suggests that the use of information theory can be generalized to analyze entire sets of marker gene data (Eren et al., 2014; Ramette and Buttigieg). The ideal result of a properly partitioned marker gene dataset will have the minimum number of units that contains minimum entropy (i.e., none of the nucleotide positions in final units will have entropy that exceeds the expected error rate of the sequencing device), which in fact can be achieved through multiple ways. Indeed, the search for algorithms that can provide single-nucleotide resolution without relying on arbitrary percent similarity thresholds is not limited to entropy-based approaches: studies that aim to address the same issue include distribution-based clustering (Preheim et al., 2013), cluster-free filtering (Tikhonov et al., 2015), Swarm (Mahé et al., 2015), and recently introduced DADA2 (Callahan et al., 2016).

Potential new directions for a more accurate depiction of microbial communities through marker gene amplicons come with new questions. What should microbial ecologists do with all the data they have generated, and plan to generate during the years to come? What are the computational and ecological issues that will need to be addressed for new methods to be more accessible in the field? Although our collection does not promise answers to these questions, we hope it will further stimulate the community of microbial ecologists and the developers of widely used software platforms to move beyond the use of OTUs that require arbitrary percent similarity cut-offs.

## Author contributions

All authors listed, have made substantial, direct and intellectual contribution to the work, and approved it for publication.

## Funding

AME was supported by the University of Chicago and the Marine Biological Laboratory collaboration award.

### Conflict of interest statement

The authors declare that the research was conducted in the absence of any commercial or financial relationships that could be construed as a potential conflict of interest.
